# The Health and Attainment of Pupils in Primary Education

**DOI:** 10.23889/ijpds.v8i2.2258

**Published:** 2023-09-14

**Authors:** Michaela James

**Affiliations:** 1 Swansea University, Swansea, United Kingdom

## Background

HAPPEN (The Health and Attainment of Pupils in Primary EducatioN) aims to empower schools to make meaningful changes by gaining a better understanding of pupil’s physical, psychological, emotional and social health. HAPPEN has had 30,000+ children across Wales take part to develop an e-cohort that is supported by public engagement and involvement.

## Methods

Pupils (aged 7-11 or years 4/5/6) complete a self-report survey within school about aspects of their health and wellbeing. HAPPEN is directly aligned to the new Curriculum for Wales and has a strong utilisation of consultation, engagement and collaboration with school staff and local authorities. Schools receive an individual report presenting group-level data on pupils’ health and wellbeing while creating a dataset of health and wellbeing across Wales. This data has been linked to routine data and has the potential to make signficant impact in Wales.

## Findings

HAPPEN data has most recently been used to explore children’s health and wellbeing during the Covid-19 pandemic. This included a retrospective cohort study using an online cohort survey (January 2018 to February 2020) linked to routine PCR SARS-CoV-2 test results to explore health-related behaviours in children (2018-2020) and association with being tested and testing positive (2020-2021). This found associations relating to parental health literacy and monitoring behaviours. As well as this, free school meal status was used as a proxy for deprivation in an exploratory study on the impact of school closures on the health and well-being. The physical health (eg, physical activity and diet) of children eligible for FSM may be affected by prolonged school closures. HAPPEN research is driven by the wants and needs of its user-group.

## Interpretation

HAPPEN exists to empower schools and practitioners to improve the health and wellbeing of children across Wales by feeding into policy and practice. This infrastructure has facilitated the creation of a dataset that can be linked to answer past and future questions including an evaluation of current free school meal roll-out.

